# Effects of Carbohydrate Counting Method on Metabolic Control in Children with Type 1 Diabetes Mellitus

**DOI:** 10.4274/jcrpe.1191

**Published:** 2014-06-05

**Authors:** Damla Gökşen, Yasemin Atik Altınok, Samim Özen, Günay Demir, Şükran Darcan

**Affiliations:** 1 Ege University School of Medicine, Department of Pediatric Endocrinology, İzmir, Turkey

**Keywords:** type 1 diabetes, blood glucose, dietary carbohydrates, medical nutrition therapy, child, adolescents

## Abstract

**Ob­jec­ti­ve:** Medical nutritional therapy is important for glycemic control in children and adolescents with type 1 diabetes mellitus (T1DM). Carbohydrate (carb) counting, which is a more flexible nutritional method, has become popular in recent years. This study aimed to investigate the effects of carb counting on metabolic control, body measurements and serum lipid levels in children and adolescents with T1DM.

**Methods:** T1DM patients aged 7-18 years and receiving flexible insulin therapy were divided into carb counting (n=52) and control (n=32) groups and were followed for 2 years in this randomized, controlled study. Demographic characteristics, body measurements, insulin requirements, hemoglobin A1c (HbA1c) and serum lipid levels at baseline and at follow-up were evaluated.

**Results:** There were no statistically significant differences between the groups in mean HbA1c values in the year preceding the study or in age, gender, duration of diabetes, puberty stage, total daily insulin dose, body mass index (BMI) standard deviation score (SDS) and serum lipid values. While there were no differences in BMI SDS, daily insulin requirement, total cholesterol, low-density lipoprotein and triglyceride values between the two groups (p>0.05) during the follow-up, annual mean HbA1c levels of the 2nd year were significantly lower in the carb counting group (p=0.010). The mean values of high-density lipoprotein were also significantly higher in the first and 2nd years in the carb counting group (p=0.02 and p=0.043, respectively).

**Conclusion:** Carb counting may provide good metabolic control in children and adolescents with T1DM without causing any increase in weight or in insulin requirements.

## INTRODUCTION

Medical nutritional therapy is one of the cornerstones of diabetes care in children and adolescents with type 1 diabetes mellitus (T1DM). It aims to provide sufficient and appropriate energy as well as macro-and micronutrients for optimal growth, development and glycemic control. Carbohydrate (carb) counting allows adjustment of the prandial insulin dose for actual carb intake in T1DM patients on intensive insulin therapy. Carbs are the primary nutrients affecting postprandial glycemic response. Therefore, by calculating the carb amounts in each meal, the insulin doses required to preserve postprandial blood glucose within normal limits can be predicted (1,2). In the past 15 years, the introduction of insulin analogs and insulin pump therapy has made carb counting more popular. To date, there are no reports of randomized controlled studies pertaining to the efficacy of carb counting in metabolic control and body measurements of children with T1DM. The aim of this study was to investigate the effect of carb counting on body measurements, glycemic control and serum lipid levels in children and adolescents with T1DM. 

## METHODS

After exclusion of patients with obesity, chronic complications and/or communication difficulties, 110 T1DM children and adolescents aged 7-18 years were enrolled in the study. The duration of diabetes was >1 years in all patients. Before the study, all patients were on the traditional exchange-based meal plan and were using glargine/detemir basal-bolus insulin regimens (fixed doses of insulin for food and changing the doses based on blood glucose levels). Out of the 110 patients, 55 were allocated randomly to the study group (the carb counting group) and 55 served as controls. Three patients from the study group who did not attend the follow-up visits regularly or could not acquire adequate carb counting skills after training were excluded, thus the study has been conducted on 52 children and adolescents with T1DM in this group. In the control group, 5 patients who withdrew their consent and 18 who did not attend the 3-month follow-up visits regularly were also excluded, thus the study has been conducted with 32 children and adolescents with T1DM in this group.

Age, weight, height, body mass index (BMI), total daily insulin requirements (U/kg/day) and HbA1c were recorded in all patients at baseline and at 3-month intervals. Additionally, morning fasting total cholesterol (TC), low-density lipoprotein (LDL), high-density lipoprotein (HDL) and triglyceride (TG) levels were measured with 1-year intervals. Height was measured to the nearest centimeter using a rigid stadiometer. Weight was measured unclothed to the nearest 0.1 kg using a calibrated balance scale. BMI was calculated by using the weight (kg)/height (m2) formula. Standard deviation scores (SDS) for weight, height and BMI were calculated using the reference values for Turkish children (3). HbA1c measurements were performed by capillary method using the Nycocard II Reader (Axis-Shield Diagnostics Ltd, Dundee, UK) device which is suitable for the International Federation of Clinical Chemistry (IFCC) Working Group reference system. Annual mean values for HbA1c were calculated by total HbA1c values divided by measurement times. TC, LDL, HDL and TG levels were measured using a Beckman Coulter Uni Cel® (Fullerton, CA) automatic analyzer.

During a 2-week program, a diabetes team consisting of a diabetologist, a dietician and a nurse trained the patients in the carb counting group to count carbs. In the first week of the training sessions, the patients learned about the biological and nutritional contents of the food groups (carbs, fats, proteins, fiber, micronutrients) and their effects on blood glucose; they also learnt how to estimate the amount of carbs per meal. In addition, the study group received information about the importance of introducing a daily amount of carbs equal to 50%-55% of the total caloric intake and about the distribution of carb between meals. In the second week, the patients learned how to manage simple carbs and snacks and to adjust insulin doses in relation to the carb content of the meals, exercise and pre meal blood glucose values. Ideal insulin requirements were defined by measuring pre- and post-prandial as well as midnight blood glucose levels. In patients who achieved normal/near-normal blood glucose values at the end of 1 week, insulin/carb ratio (I/C) and insulin sensitivity factor (ISF) were calculated individually by dividing total daily insulin amounts to 500 and 1800 coefficients, respectively (4). In patients who had adequate knowledge and who also applied the procedures correctly, nutrition according to calculation model was initiated. In the first month after the training, I/C and ISF values were corrected if necessary according to blood glucose follow-ups by weekly phone calls or hospital visits. Training levels and applications by patients were evaluated during the outpatient follow-up visits performed at 3-month intervals by the same dietician and pediatric endocrinologist. Training was repeated for patients as required. In the control group of patients, nutritional and diabetic educations were repeated at the baseline of study, outpatient follow-up visits were performed with 3-month intervals and the education was repeated if necessary.

The authors confirmed in writing that they have complied with the World Medical Association Declaration of Helsinki regarding ethical conduct of research involving human subjects and/or animals. The study was approved by the local ethics board and informed consent was obtained from the families of all patients.

**Statistical Analysis**

SPSS 15.0 for Windows software program was used for statistical assessments. Descriptive statistics were given as cross-tables for categorical variables, means and as standard deviations for numerical variables. Chi-square test was used for independent group comparisons in categorical variables. If distribution was normal, then paired group comparisons of numerical variables were performed with t-test, otherwise Mann-Whitney U-test was used. Repetitive ANOVA test was used for repetitive measurements. Level of significance was defined as p<0.05.

## RESULTS

The mean age of the carb counting group (n=52) was 16.4±4.5 years and that of the control group (n=32) was 17.0±5.0 years. There was no statistically significant age and gender difference between the groups and the distributions were equivalent (p=0.545, p=0.428).

Duration of diabetes, pubertal stage, mean HbA1c values during the year preceding the study, total daily insulin dose (U/kg/day), BMI, BMI SDS, as well as TG, TC, HDL and LDL values in the control and carb counting groups were similar at baseline. Demographic, clinical and laboratory characteristics of the patients at baseline are given in Table 1.

Although BMI, BMI SDS, total daily insulin doses and serum mean LDL levels showed an annual increase in the follow-up of both the control and carb counting groups (p<0.05), these changes did not differ between the groups (p>0.05). Mean HDL levels were significantly high at the end of the first and 2nd years in the carb counting group compared to the controls (p=0.02 for year 1 and p=0.043 for year 2) (Table 2).

While there was no difference between the control and carb counting groups in mean values of HbA1c at the end of the first year (8.01±1.20% and 7.58±0.97%, respectively; p=0.118), the mean values of HbA1c at the end of the second year were significantly lower in the carb counting subjects (controls vs. carb counters, 8.76±1.77% vs. 7.87±1.38%, respectively, p=0.010). When the groups were evaluated within themselves for changes in HbA1c values, there was no significant difference in mean HbA1c values 1 year before and during the study in the control group (p=0.218). However, although the absolute decrease in HbA1c was 0.42 at the end of the first year compared to baseline (from 8.43% to 8.01%; p>0.05), an absolute increase of 0.33 at the end of the 2nd year was observed compared to baseline (from 8.43% to 8.76%; p>0.05). In the carb counting group, there was a statistically significant decrease in mean HbA1c values when the values of the past 1 year at baseline and the values of the first and second years of the study were compared (p=0.024). In the carb counting group, an absolute decrease in mean HbA1c values of 0.52 was found at the end of the first year, which was statistically significant (from 8.10% to 7.58%; p=0.08). Although HbA1c value was lower at the end of the second year compared to the values before the study, the difference was not significant (HbA1c in the year preceding the study: 8.10±1.00%, HbA1c at the end of the 2nd year: 7.87±1.38; p>0.05) and there was an absolute increase of 0.29 in mean HbA1c value at the end of the second year compared with the first year (from 7.58% to 7.87%; p>0.05) (Table 3).

## DISCUSSION

Currently, carb counting is a nutritional strategy that allows a greater adherence to dietary management and consumption of a greater variety of foods for patients with DM. It also requires greater adherence by the patient to maintain an adequate blood glucose monitoring and the ability to determine the amount of carbs in the meals (1,2,5).

In this prospective randomized controlled study, we investigated the effects of carb counting method on body measurements as well as on glycemic and metabolic control in children and adolescents with T1DM. We could not find in the literature any randomized controlled study on the efficacy of carb counting in childhood. Dias et al (5) showed a decrease in HbA1c levels and an increase in daily insulin requirement at the end of the 3rd month after carb counting in 51 T1DM patients of ages 10-60 years; they did not have a control group and the follow-up period was short. Laurenzi et al (6) conducted a randomized controlled study on adult patients using insulin pumps to investigate the effects of carb counting and demonstrated a statistically significant decrease in BMI at the end of the 24th week and a significant decrease in HbA1c values (p=0.05); life quality in these patients was not different from that of the control group. Goksen et al (7) reported that among patients who use glargine insulin with two different nutritional models (exchange based meal plan and carb counting), diabetes-related worries were decreased in carb counting group, but there was no difference in HbA1c changes according to nutrition model, although a statistically significant decrease in HbA1c values was noted in both groups after 6 months. In our study, HbA1c decreased more markedly in the carb counting group, although there was no statistically significant difference in mean HbA1c values either in the year preceding the study or at the end of the first year of the study between the control and carb counting groups. When groups were evaluated within themselves in terms of HbA1c changes, we have demonstrated that carb counting method was more effective in improving HbA1c levels (Table 3). Thus, the results of our study show that carb counting method has positive effects on metabolic control in children and adolescents with T1DM. This may be related to a more flexible nutrition of these patients and to more frequent insulin injections between the main meals.

On the other hand, while the changes in TG and TC levels in the carb counting group did not differ significantly from the controls, there was a significant difference in LDL and HDL levels between the two groups at all time points during the follow-up. The increase in HDL levels might be due to increased knowledge of patients in carb counting group on healthy and balanced diet. It has been reported in two previous studies (5,8) that lipid levels are not affected by the carb counting. Further studies should be performed about this subject.

We demonstrated that daily insulin requirements were Some clinicians believe that obesity may occur due to more flexible and frequent feeding with carb counting method (1,9). In our study, BMI and BMI SDS values have shown an increase, however, there was no difference between the groups (p>0.05). We thought that the increases noted in BMI and BMI SDS values may have been related to changes in growth occurring during puberty progression. It has been demonstrated in previous studies that BMI did not increase with carb counting in children and adults and even a decrease has been shown to occur in some studies (1,5,6,10,11).

We demonstrated that daily insulin requirements were increased in both groups. The increases in insulin requirements compared with baseline values were comparable in the two groups. We suggest that the increase in insulin requirement is related to growth and puberty progression and is in line with the noted increase in BMI values, rather than being related to carb counting. Dias et al (5) reported that there was a significant increase in insulin requirement with carb counting at the 3rd month. In another adult study, it has been demonstrated that short-acting insulin requirements decreased at the end of 9 months with carb counting (11). 

Accuracy of knowledge about this nutritional method and its application by the patients and their families is critically important for the efficacy of carb counting. Mehta et al (12) conducted a study on children aged between 4 and 12 years and demonstrated that HbA1c values in patients with satisfactory knowledge on this method were 0.8% lower than those in their counterparts. In another study conducted on adolescents with T1DM aged between 12 and 18 years, it has been shown that only 23% of the subjects could count carb correctly (13). We have not used any scale to measure the knowledge of patients or their families in our study. However, knowledge and application performances of the patients and their families about carb counting have been evaluated weekly in the first month and every 3 months thereafter by a physician and dietician and missing information has been completed.

One of the limitations of our study group is the number of patients. Further large-scale studies with longer durations are required on these issues.

In conclusion, with carb counting, which is a more flexible nutritional model, better metabolic control can be obtained in children and adolescents with T1DM without causing an increase in weight and insulin requirements. Moreover, increases in HDL levels noted in these patients may serve to decrease cardiovascular disease risk. 

## Figures and Tables

**Table 1:**
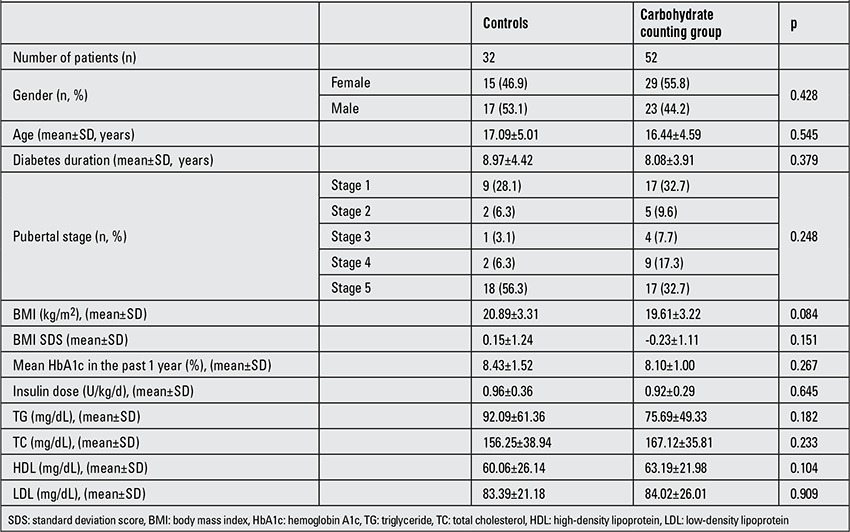
Demographic, clinical and laboratory characteristics of patients at baseline

**Table 2:**
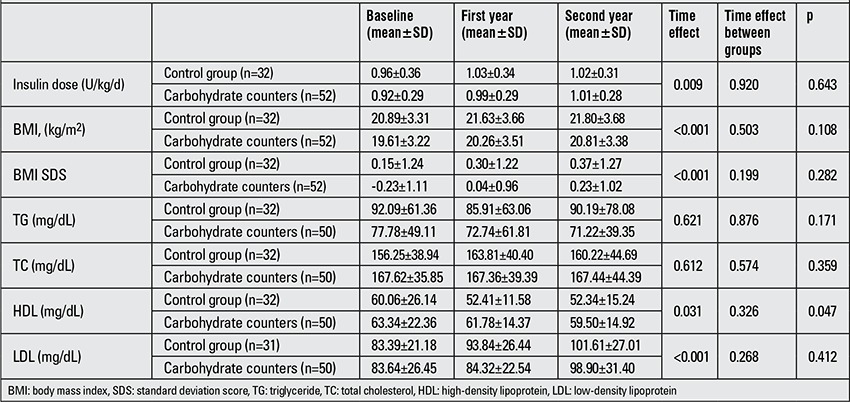
Insulin dose, BMI, BMI SDS and serum lipid values in the carbohydrate counting and control groups at baseline and during follow-up

**Table 3:**
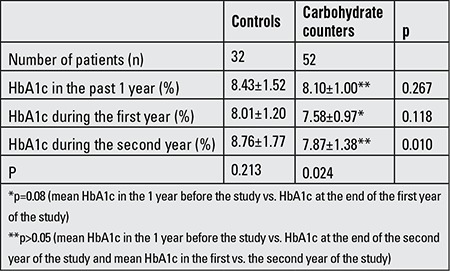
Hemoglobin A1c (HbA1c) changes in the study and control groups. HbA1c values are given as means±SD
